# Comorbid Conditions of Ehlers–Danlos Syndromes and Vulvodynia: A Latent Class Analysis ^✩,✩✩^

**DOI:** 10.1016/j.pmn.2026.03.005

**Published:** 2026-04-10

**Authors:** Jennifer E. Glayzer, Bethany C. Bray, William H. Kobak, Caleb M. Trujillo, Crystal L. Patil, Hongjin Li, Clair A. Francomano, Judith M. Schlaeger

**Affiliations:** * Department of Human Development Nursing Science, University of Illinois Chicago College of Nursing, Chicago, IL; † Center for Clinical and Translational Science, University of Illinois Chicago, Chicago, IL; § Department of Obstetrics and Gynecology, University of Illinois Chicago College of Medicine, Chicago, IL; ║ University of Washington Bothell School of Interdisciplinary Arts and Science, Seattle, WA; ¶ Department of Health and Biological Sciences, University of Michigan College of Nursing, Ann Arbor, MI; # Department of Medical and Molecular Genetics, Indiana University College of Medicine, Indianapolis, IN

**Keywords:** Dyspareunia, Vulvodynia, Ehlers–Danlos syndromes, Latent class analysis, Hypermobility Spectrum Disorders

## Abstract

**Purpose:**

Having an Ehlers-Danlos syndrome (EDS) or hypermobility spectrum disorder (HSD) may increase the likelihood of vulvodynia six-fold. Studying vulvodynia in EDS/HSD may help identify causes of vulvodynia and potential treatments. Currently there are no consistently effective treatment methods for vulvodynia. We aim to identify comorbid condition patterns and how they affect a participant’s likelihood of screening positive for vulvodynia.

**Design:**

Online survey.

**Methods:**

A secondary analysis was conducted using latent class analysis on data from females aged ≥18 years with EDS or HSD ( *N* = 1,016) who were screened for vulvodynia.

**Results:**

Five comorbid condition patterns of 9 indicators were identified. Among the patterns 2 overarching comorbid condition phenotypes were present, 1) non-musculoskeletal phenotype ( *n* = 185) comprised of mast cell activation disorder, gastrointestinal conditions, and dysautonomia; and 2) pain phenotype ( *n* = 442) comprised of chronic pain and pelvic instability; 201 participants endorsed both phenotypes. The non-musculoskeletal phenotype incurred the smallest likelihood of screening positive, followed by the pain phenotype. Endorsed both phenotypes resulted in significantly greater likelihood of screening vulvodynia positive ( *p* < 0.05) compared to one phenotype.

**Conclusions:**

Phenotypes may constitute 1) different pathways for developing vulvodynia and/or 2) different subtypes of vulvodynia and/or EDS. We hypothesize that as a person accumulates comorbid conditions, their allostatic load increases, and once a personal allostatic load threshold is crossed, vulvodynia may develop.

**Clinical Implications:**

Different pathways for developing vulvodynia may explain why there is no consistently effective treatment for vulvodynia. Phenotypes may be able to be used to develop personalized treatment methods.

Vulvodynia is vulvar pain, lasting for at least three months, and may have other potentially associated symptoms; including dyspareunia (pain during sexual penetration) (Bornstein et al., 2016). Pain can be so severe that those that it affects are unable to wear tight clothing that contacts the vulva (Schlaeger et al., 2023) and may consider suicide due to the pain (Young & Miller, 2019). The cause(s) of vulvodynia is unknown, and there are no known consistently effective treatments (Bergeron et al., 2020; Bornstein et al., 2016; Goldstein et al., 2016; Haefner et al., 2005; Schlaeger et al., 2023; Torres-Cueco & Nohales-Alfonso, 2021; Wesselmann et al., 2014). Factors associated with vulvodynia are central nervous system sensitization, likely related to co-morbid chronic pain conditions (Giesecke et al., 2004; Torres-Cueco & Nohales-Alfonso, 2021); neuroinflammatory proliferation (Krapf et al., 2022) related to exaggerated immune response from recurrent infections, allergic reactions and/or increased presence of mast cells (Bornstein et al., 2022; Krapf et al., 2022); pelvic floor dysfunction; headaches, including temporomandibular joint disorder and migraines; irritable bowel syndrome; interstitial cystitis; psychological factors like anxiety and depression (Schlaeger et al., 2023); and use of combined oral contraceptives (Bergeron et al., 2020; Bornstein et al., 2016, 2022; Schlaeger et al., 2023). However, it is unknown how these factors are related to or interact with vulvodynia, or each other (Bergeron et al., 2020; Bornstein et al., 2016; Goldstein et al., 2016; Haefner et al., 2005; Schlaeger et al., 2023; Wesselmann et al., 2014). Current treatment methods that target these factors associated with vulvodynia include oral tricyclic antidepressant, serotonin norepinephrine reuptake inhibitors (SNRIs), selective serotonin reuptake inhibitors (SSRIs), and antiepileptics to target chronic pain and central nervous system sensitization; diazepam, botulinum toxin A, physical therapy, dilators, transcutaneous electrical nerve stimulation (TENS), and biofeedback to tareget pelvic floor dysfunction; and topical estradiol and testosterone to address hormonal components (Schlaeger et al., 2023). Other treatment methods include acupuncture, cognitive behavioral therapy, and topical lidocaine. A multimodal treatment approach has been found to be the most effective treatment method.(Schlaeger et al., 2023) The lack of consistently affective treatment methods and a multimodal treatment approach being the most successful for treating vulvodynia may suggest multiple pathways for developing vulvodynia, with no one treatment method able to address all pathways.

It is suspected that 50% of females who have an Ehlers–Danlos syndrome (EDS) or hypermobility spectrum disorder (HSD) may have vulvodynia, six times the rate of the general population (Glayzer et al., 2021). EDS are a group of 13 genetic connective tissue disorders that cause fragile lax connective tissue (Blackburn et al., 2018; Malfait et al., 2017) resulting in joint hypermobility; skin fragility; defective scarring; vascular and internal organ fragility prone to ruptures, gastroparesis, dysautonomia, and prolapse (Castori, 2016; Castori et al., 2015; Malfait et al., 2017; B. Tinkle et al., 2017; B.T. Tinkle & Levy, 2019). All EDS types, except for hypermobile EDS (hEDS), are diagnosed with genetic testing (Blackburn et al., 2018; Malfait et al., 2017). Since the gene(s) responsible for hEDS have not been identified, hEDS is diagnosed based on clinical presentation including the manifestations listed above (Malfait et al., 2017). People with HSD present with joint hypermobility and significant comorbidities that incur high symptom burden (Tinkle & Levy, 2019; Voermans et al., 2010; Voermans & Knoop, 2011; Volberding et al., 2022), similar to hEDS, but do not meet the diagnostic criteria for hEDS. People with EDS and hEDS have frequent debilitating injuries from activities of daily living due to unstable joints (Malfait et al., 2017; Rombaut et al., 2011; Tinkle et al., 2017; Voermans & Knoop, 2011), such as putting on and removing shoes or brushing one’s hair. These injuries result in chronic pain which typifies hEDS and HSD resulting in central nervous system sensitization (Castori, 2016; Rombaut et al., 2015) with 87% of people with EDS reporting difficulty completing activities of daily living due to pain (Voermans et al., 2010).

Many of the factors associated with vulvodynia are also associated with EDS (Glayzer et al., 2021). Investigating people with EDS and vulvodynia, and identifying what characteristics make people with EDS more likely to have vulvodynia, may help further understanding of vulvodynia. In a sample of females who have either EDS or HSD, we aimed to 1) identify patterns of comorbid conditions in two groups of people, those that screened positive for vulvodynia (vulvodynia+) and those who screened negative (vulvodynia−) and 2) examine the relationship between those co-morbid condition patterns and a person’s likelihood of screening vulvodynia+. Comorbid condition patterns made up of factors associated with vulvodynia and/or comorbidities of EDS can identify how the cooccurrence of specific comorbid conditions, rather than a single condition or count of comorbid conditions, impact a person’s likelihood of screening vulvodynia+. This information can be used to help identify new comorbid conditions associated with vulvodynia, shed light on the pathophysiology underlying vulvodynia, and determine why no consistently effective treatment method has been identified. Examining the likelihood of a person having vulvodynia based on comorbid condition patterns provides a pragmatic, holistic, and nuanced approach to studying vulvodynia, which better represents the clinical presentation of patients.

## Methods

**Figure 1.**
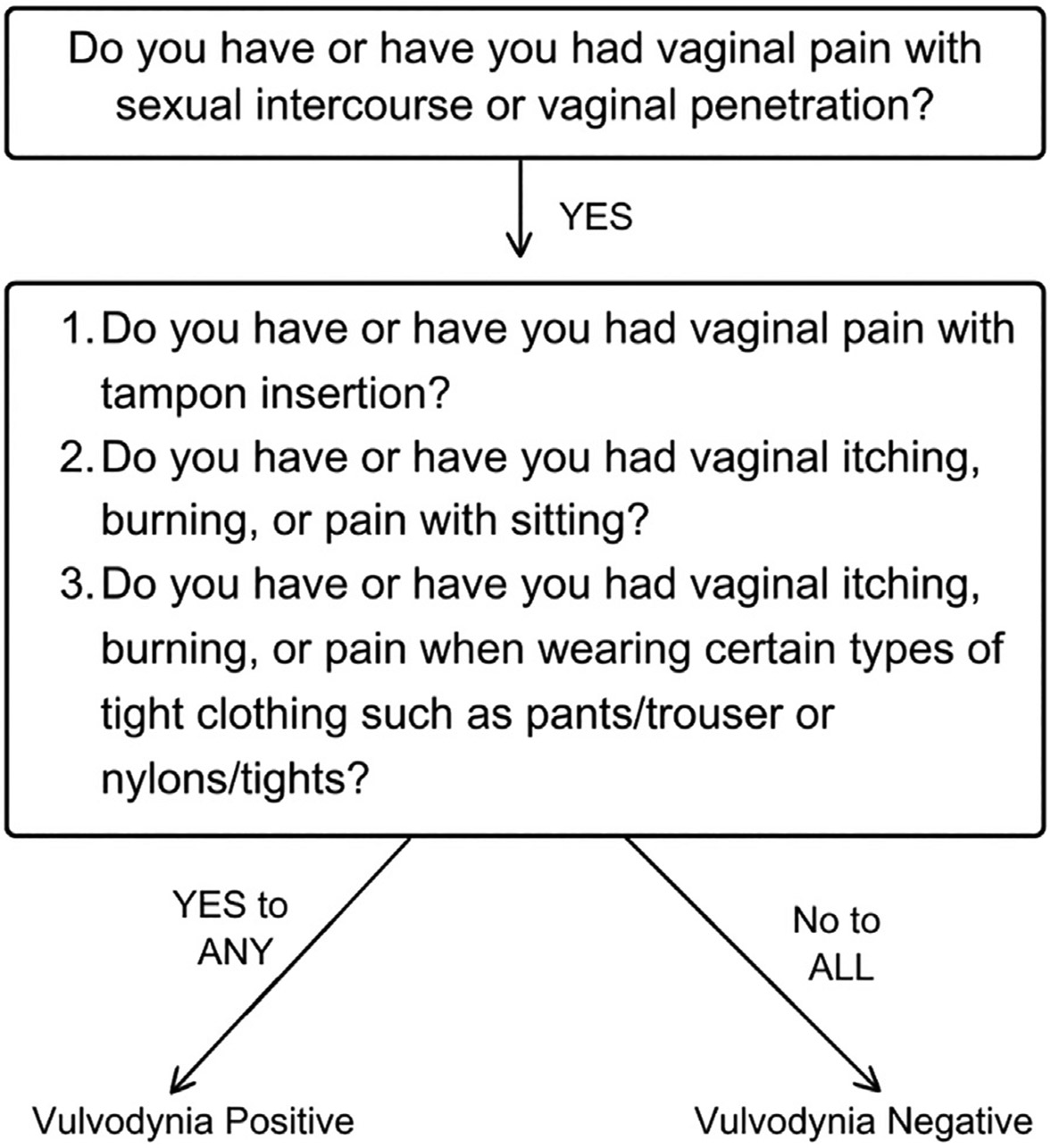
Vulvodynia screening tool [9].

This study is a secondary analysis of data (Glayzer et al., 2021) produced from, *High rates of dyspareunia and probable vulvodynia in Ehlers–Danlos syndromes and hypermobility spectrum disorders: An online survey* (Glayzer et al., 2021). Recruitment methods for this study are described in, *Lack of Diversity in Research on Females with Ehlers–Danlos Syndromes: Recruitment Protocol for a Quantitative Online Survey* (Glayzer et al., 2024). This online, cross-sectional survey was conducted using Qualtrics (Qualtrics^®^, Provo, UT) from June to July 2019. The study was approved by the University of Illinois Chicago Institutional Review Board, protocol number 2019-0219. People with EDS or HSD were consulted during the development of the survey to ensure the language used was in line with the community’s values. Potential participants were recruited from EDS and HSD support groups on Facebook (Meta, Menlo Park, CA). Participants read and agreed to participate by signing an electronic consent. No personal identifiable information was collected from participants. Inclusion criteria were: 1) a self-reported diagnosis of EDS (any type) or HSD previously confirmed by a healthcare provider; 2) assigned to the female sex at birth and had not had genital gender reassignment surgery; 3) 18 years of age or older; and 4) able to read English. Self-reported EDS diagnosis is an accepted inclusion criterion within the EDS research community (Arthur et al., 2016; Ashtari & Taylor, 2022; Bennett et al., 2021; Chan et al., 2019; Williams et al., 2023) and was used to obtain a large globally diverse sample that would not be possible if a participant’s diagnosis needed to be confirmed via medical evaluation of medical records. Participants were screened for vulvodynia using four questions that were found to be valid and reliable (Fig. 1) (Harlow et al., 2009; Reed et al., 2006). Understanding that a screening tool is used to identify people who may be at risk for having a condition but need further evaluation before they can be diagnosed, this four question screening tool can be used to screen for vulvodynia but not diagnose vulvodynia, which requires a pelvic exam. Therefore, participants are described as screening positive (vulvodynia+) or negative (vulvodynia−) as opposed to being diagnosed with vulvodynia. Vulvodynia screening status was used as an outcome variable in the latent class analysis (LCA).

**Figure 2.**
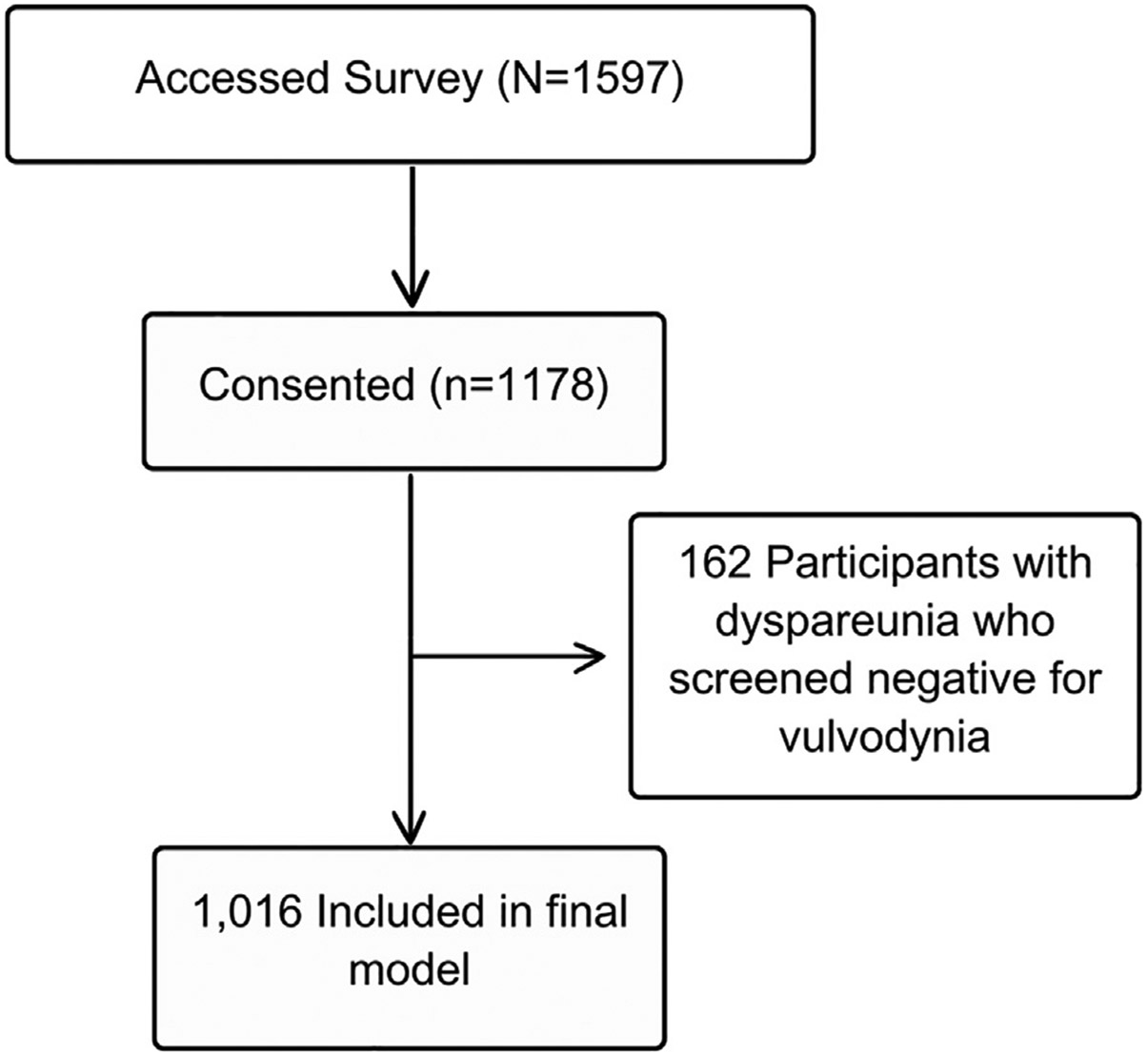
CONSORT flow chart.

Figure 2 shows a CONSORT flow chart with 1,016 participants being included in the analysis. The online survey was accessed by 1,597 potential participants, of which 1,178 consented, and 1,016 were included in the analysis (Glayzer et al., 2021). Of the 1,178 participants, 162 were excluded because they had dyspareunia but did not screen positive for vulvodynia. These participants were excluded because 1) this group had a small sample size compared to those who were a) vulvodynia+ or b) vulvodynia− without dyspareunia, and 2) dyspareunia was part of the screening tool for vulvodynia resulting in collinearity with those who were vulvodynia+. All participants included in the analysis completed the survey with no missing data. Eleven different types of EDS are represented in the analysis, 91% had hEDS or HSD. Among people with EDS in the general population, 80%-90% have hEDS. The average age of participants was 37.9. Although age related factors, such as hormonal changes during menopause, can affect vulvar pain experiences, there was no significant difference in age between those who screened vulvodynia+ or vulvodynia−; therefore age was not included in the analysis. When asked about their racial and ethnic identities, the majority identified as White (92.4%) with 3.6% identifying as Hispanic or Latino. One participant identified as transgender. See Table 1 for additional participant demographics.

### Latent Class Indicators

A LCA was used to identify comorbid condition patterns in females who have been diagnosed with EDS or HSD. EDS comorbid conditions and factors associated with vulvodynia such as central nervous system sensitization, exaggerated immune response, pelvic floor dysfunction, headaches, irritable bowel syndrome, and interstitial cystitis were included in the analysis as indicators. The total number of comorbid conditions and vulvodynia-associated factors was too large for the analysis due to sparseness of data. To conduct a robust LCA, comorbid condition categories, referred to as indicators, were used instead of individual comorbid conditions.

A total of nine indicators were included in the LCA: dysautonomia, neck-related conditions, deep penetration dyspareunia, chronic pain, pelvic instability, gastrointestinal conditions, vulvar skin conditions, endometriosis, and mast cell activation disorder (MCAD). Indicators were chosen from comorbid conditions collected via self-report from the online survey. Comorbid condition data were reported by participants using a self-reported checklist of conditions, fill-in-the-blank responses, and questions that inquired about specific conditions. Conditions that were written in the fill-in-the-blank portion were assessed by content experts (JG and JS). Conditions that occurred at a low frequency of less than 10% and were not known to be associated with vulvodynia or EDS or HSD were excluded. Thirty-two conditions met these criteria and were comprised of EDS comorbidities, factors associated with vulvodynia, and comorbid conditions that cause dyspareunia and vulvar pain.

The list of 32 conditions was condensed into the nine indicators by collapsing similar comorbid conditions into categories based on symptoms, body systems affected, and/or pathology. The analysis was also conducted with 14 indicators that included conditions related to EDS or vulvodynia and had a frequency near the cutoff frequency of 10%, like autoimmune conditions. We found the additional five indicators were too sparse to draw meaningful conclusions. Conditions that were subtypes of a larger umbrella condition were collapsed into one comorbid condition type. For example, small fiber neuropathy and peripheral neuropathy were combined into the neuropathy category. Headache subtypes, craniocervical instability, Chiari malformation, and temporomandibular joint dysfunction were combined since they can all cause headaches and have overlapping symptoms. Gynecological conditions of untreated vaginal or cervical infections, vulvar skin diseases, and vulvar lacerations were combined since they are all conditions that may be ruled out to establish a diagnosis of vulvodynia. Pelvic inflammatory disease, polycystic ovarian syndrome, and uterine cysts and fibroids were collapsed into a deep dyspareunia category. Endometriosis was retained as an individual indicator because it was reported by more than 10% of participants, thereby mitigating concerns regarding data sparsity. Moreover, endometriosis represents a primary pelvic pain disorder (Agarwal et al., 2019), whereas the conditions categorized under deep dyspareunia are not inherently pelvic pain disorders but may elicit pain upon contact with affected areas during penetration and occurred at frequencies below 10%. Pelvic instability and chronic pain were identified as two separate indicators. Both indicators may be connected to vulvodynia through central nervous system sensitization. For example, one symptom of pelvic instability is pain (Morin et al., 2017) and chronic pain may lead to central nervous system sensitization (Latremoliere & Woolf, 2009; Ossipov et al., 2010; Woolf, 2011). However, for this analysis, chronic pain and pelvic instability were kept separate because pelvic floor dysfunction due to pelvic instability, and central nervous system sensitization due to chronic pain are both individually significant factors associated with vulvodynia (Cox & Neville, 2012; Schlaeger et al., 2023; Morin et al., 2017) and therefore represent different concepts. The full list of conditions, their frequencies, how each condition was defined, how conditions were combined into categories, and the reasons conditions were excluded can be found in the supplemental material.

### Data Analysis

The nine indicators identified were used to conduct an LCA. The LCA detected and described the size and characteristics of classes of participants with unique comorbid conditions patterns based on the nine indicators (Collins & Lanza, 2010). Maximum likelihood estimation procedures were used to derive two sets of parameter estimates: latent class membership probabilities and item-response probabilities for each latent class. Latent class membership probabilities represent the prevalence of each comorbid-condition pattern in the sample. Each item-response probability represents the likelihood of participants having an indicator (comorbid condition), given membership in a class (comorbid-condition pattern). The probability an indicator was endorsed was used to interpret the classes. All analyzes were conducted with Latent Gold 6.0 (Statistical Innovations Inc, Arlington, MA).

### Model selection

The number of classes (comorbid condition patterns) in the model were chosen based on model fit and theoretical interpretability. Latent class models with one to six classes were considered. Several model-fit indices, including penalized-fit-criteria Akaike Information Criterion (AIC), Bayesian Information Criterion (BIC), and sample-size-adjusted Bayesian Information Criterion (aBIC), were compared across competing models. The optimal model was selected based on how small the AIC, aBIC, and BIC fit indices were, class homogeneity and separation, model interpretability, and parsimony.

### Vulvodynia screening

Vulvodynia screening status was used as an outcome variable. Age-related factors such as hormonal changes during menopause can influence vulvar pain experiences. However, there was no significant difference in age by vulvodynia screening status; therefore, age was not included as a correlate in the LCA. Similarly, race, ethnicity, and country of origin did not significantly differ by vulvodynia screening status and were also excluded as correlates in the LCA. Measurement invariance testing was used to ensure the structure or make-up of the classes (comorbid condition patterns) were the same for those who screened vulvodynia+ and vulvodynia−. The likelihood ratio test was significant between 1) the model where classes were different in those who screened vulvodynia+ compared to vulvodynia−, and 2) the model where classes were the same in people who screened vulvodynia+ and vulvodynia−. However, the AIC, BIC, and aBIC suggested the invariant model, where class structure was the same across vulvodynia screening status, provided a better balance between fit and parsimony. Manual inspection of the variant and invariant models suggested limited loss of information when measurement invariance was imposed. Thus, vulvodynia screening was added as an outcome variable; class (comorbid condition pattern) structures were not allowed to vary across vulvodynia screening results and the class structure was the same for both those who screened vulvodynia+ and vulvodynia−. Model fit information for the invariant and variant models is presented in Table 2. The likelihood of screening vulvodynia+ was compared across classes to understand how a participant’s membership in a particular class impacted their likelihood for screening vulvodynia+.

## Results

### Latent Class Structure and Vulvodynia Likelihood

**Figure 3.**
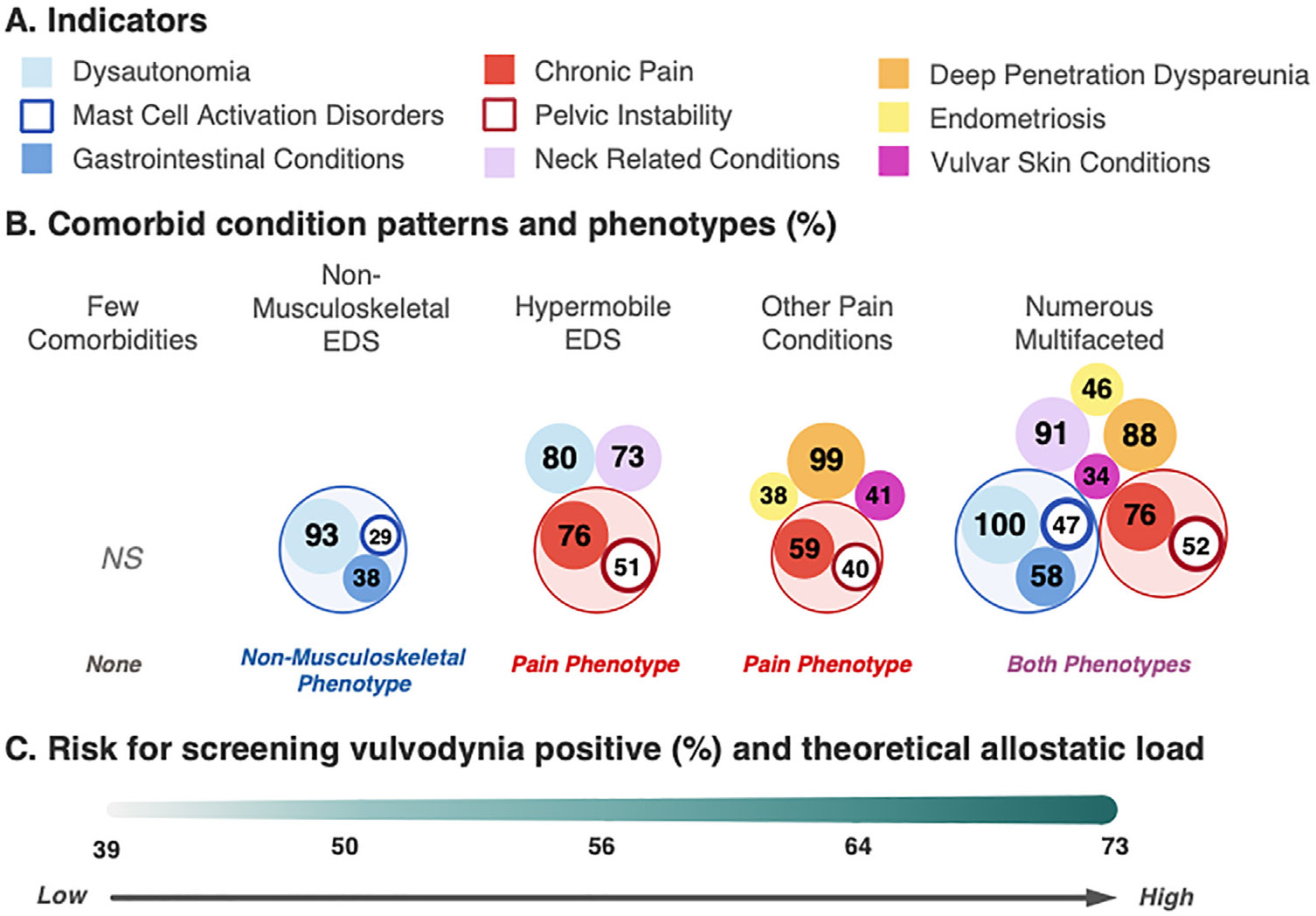
Visual representation of comorbid condition patterns and phenotypes and their impact on vulvodynia probable screening status. Figure 3. Comorbid condition patterns and their impact on participants’ likelihood of screening positive for vulvodynia A visual representation of the data in Table 4. Indicators in section A are the nine comorbid condition categories included in the latent class analysis. Section B shows the 5 classes of comorbid condition patterns identified using the 9 indicators. The name of each class is shown above the corresponding circle cluster. Each circle represents the percentage of participants that endorsed the respective indicator given class membership. The large blue and red circles that encapsulate indicators represent the phenotypes. The phenotypes present in each class are listed below the circle clusters. The first arrow in section C demonstrates the increased likelihood of screening vulvodynia+ from one cluster and/or phenotype to another. The second arrow represents our hypotheses that allostatic load increases from one class and/or phenotype to the next in the order depicted, and that as allostatic load increases, likelihood of screening vulvodynia+ also increases.

Figure 3 shows the comorbid condition pattern for each class and the probability of members in each class screening vulvodynia+ or vulvodynia−. Table 3 presents the model fit statistics for the LCA models with three to five class solutions. The BIC suggested a three-class model, aBIC suggested a four-class model, and AIC suggested a five-class model. Comorbid condition patterns in models with three and four classes had low homogeneity and poor separation, resulting in class structures that lacked unique patterns of comorbid conditions, which limited theoretical interpretations. Thus, the five-class model, shown in Table 4, was selected as optimal for further analysis.

Table 4 includes model details for the chosen model, including all indicators, class prevalences, and the likelihood of class members screening vulvodynia+ with statistical differences between the classes. Members of class one ( *Few Comorbidities,* 18.5%) reported few, if any, of the comorbid conditions. Members of class two ( *Non-musculoskeletal EDS Comorbidities,* 18.3%) were more likely to have non-musculoskeletal comorbid conditions associated with EDS. Members of class three ( *Hypermobile EDS Comorbidities,* 17%) were more likely to have comorbid condition indicators associated with specifically hEDS and HSD. Members of class four ( *Other Pain Conditions*, 26.5%) were more likely to have comorbid conditions that are associated with vulvar pain, dyspareunia, and/or vulvodynia. Members of class five ( *Numerous Multifaceted Comorbidities*, 19.8% prevalence) were more likely to have all comorbid conditions than participants in any other class.

While the likelihood of screening vulvodynia+ increased as the number of comorbid conditions (indicators) endorsed increased, the likelihood of screening vulvodynia+ was based on the pattern of comorbid conditions endorsed and not the overall number of conditions a participant endorsed. Comorbid conditions (indicators) endorsed by class were qualitatively different, meaning participants were not moving from a low-likelihood class to a high-likelihood class through the acquisition of additional comorbid conditions. This is because the class (comorbid condition pattern) that was endorsed did not overlap, and conditions included in the analysis are not conditions that naturally resolve like chronic pain or endometriosis.

When examining the makeup of classes (comorbid condition patterns) and the class likelihood of screening vulvodynia+, two phenotypes emerged: pain and non-musculoskeletal. Figure 3 shows the indicators in each class and the phenotypes present in each class. The pain phenotype includes the co-occurrence of the chronic pain and pelvic instability indicators. The non-musculoskeletal phenotype includes the co-occurrence of mast cell activation disorder, gastrointestinal conditions, and dysautonomia. Dysautonomia is a broad term used to describe a wide range of autonomic impairments, including dysregulation of blood pressure and heart rate, which can result in dysfunction of the neural axis leading to fatigue, dizziness, fainting, or multiple system atrophy, and is a comorbid condition of EDS (Hovaguimian, 2023; Mathias et al., 2021). The conditions in the non-musculoskeletal phenotype all have overlapping symptoms; with MCAD and dysautonomia sharing the symptoms of weakness, fatigue, functional gastrointestinal disorders, hypotension, exercise intolerance, impaired cognition, headaches, and palpitations (Brock et al., 2021; Doherty & White, 2018; Kucharik & Chang, 2020; Mathias et al., 2021; Theoharides et al., 2024; Wu & Ho, 2024). Gastrointestinal issues are a symptom of both dysautonomia and MCAD. It is recognized that the symptoms of MCAD and dysautonomia can exacerbate one another, it is unclear how both these conditions impact each other, and further research needed (Brock et al., 2021; Doherty & White, 2018; Kucharik & Chang, 2020; Mathias et al., 2021; Theoharides et al., 2024; Wu & Ho, 2024).

Members of the *Few Comorbidities* class did not endorse either phenotype and had the lowest likelihood of screening vulvodynia + (39%). Members of the *Non-musculoskeletal Comorbidities* class endorsed the non-musculoskeletal phenotype and had a higher likelihood of screening vulvodynia+ (50%) than the *Few Comorbidities* class; however, this difference was not significant ( *p* = .24). Participants who endorsed the non-musculoskeletal phenotype had a lower likelihood of screening vulvodynia+ than those who endorsed the pain phenotype. The pain phenotype was present in both the *Hypermobile EDS Comorbidities* class (56%) and the *Other Pain Conditions* class (64%). Those in the *Other Pain Conditions* class had a significantly higher likelihood of screening vulvodynia+ *(p* = *.005)* than those in the non-musculoskeletal phenotype, whereas those in the *Hypermobile EDS Comorbities* class also had a higher liklihood, but this was not statisically significant. While members of both the *Hypermobile EDS Comorbidities* class and the *Other Pain Conditions* class endorsed the pain phenotype, those in the *Other Pain Conditions* class also endorsed endometriosis, an additional pain condition that may explain the increased likelihood of screening vulvodynia+ in the *Other Pain Conditions* class comapred to the *Hypermobile EDS Comorbidities* class. Members of the *Numerous Multifaceted Comorbidities* (73%) class endorsed both the pain and non-musculoskeletal phenotypes and had the highest likelihood of screening positive for vulvodynia, significantly higher than those who endorsed the *Other Pain Conditions* class ( *p* = .02). Having participants who endorse both phenotypes is akin to having more than one pain type concomitantly (e.g. nociceptive, neuropathic, nociplastic, and/or mixed pain) (Latremoliere & Woolf, 2009; Orhurhu et al., 2023; Si et al., 2024).

The non-musculoskeletal phenotype did not contribute to a significant likelihood of screening positive for vulvodynia. However, when added to the pain phenotype the combined likelihood of screening positive for vulvodynia was significantly greater than the pain phenotype alone indicating the non-musculoskeletal phenotype does play a role in the development of vulvodynia. The co-occurrence of both phenotypes resulted in significantly higher likelihood of screening positive for vulvodynia than either of the phenotypes individually.

## Discussion

### Vulvodynia Findings

Vulvodynia may have numerous causative factors and be a symptom rather than a disease state. We identified phenotypes that may differently affect a person’s likelihood for vulvodynia and represent separate subtypes and/or pathways for developing vulvodynia. Endorsement of both phenotypes by some participants suggests both subtypes and/or pathways can occur simultaneously, similar to having multiple types of pain (Latremoliere & Woolf, 2009; Orhurhu et al., 2023; Ossipov et al., 2010; Si et al., 2024). People who endorse both phenotypes have the highest likelihood of screening vulvodynia+. The lack of consistently effective treatments for vulvodynia may be explained by qualitatively different phenotypes. While our study was a cross-sectional analysis that does not determine causal relationships, phenotypes may be used as a starting point to determine causative etiologies and development of new treatment methods. Persons with the pain phenotype may respond better to treatments targeting central nervous system sensitization. There are no vulvodynia treatments that address conditions in the non-musculoskeletal phenotype which may explain why certain people do not respond to current treatment methods. Mast cell stabilizers may treat this group since mast cells are associated with vulvodynia and are part of the non-musculoskeletal phenotype.

Two studies (Nguyen et al., 2013; Reed et al., 2012) have used LCA or cluster analysis to examine comorbid conditions and vulvodynia without EDS. These studies characterized vulvodynia but did not evaluate likelihood of having vulvodynia. Nguyen et al. (2013) found classes of 1) no conditions endorsed and 2) at least one condition endorsed. Reed et al. (2012) found classes of 1) only one condition endorsed and 2) multiple conditions endorsed (Reed et al., 2012). Both studies included comorbid conditions similar to this study. Neither Nguyen et al. (2013) nor Reed et al. (2012) identified dysautonomia as a condition that increases the likelihood of having vulvodynia.

We identified dysautonomia as a factor that increased the likelihood of screening vulvodynia+. Both dysautonomia and central nervous system sensitization are a dysregulation or dysfunction of the nervous system, thought to be due to a prolonged exposure to a stressor (Goldstein, 2020; Latremoliere & Woolf, 2009). Central nervous system sensitization is related to prolonged exposure to nociceptive input (Ji et al., 2018; Latremoliere & Woolf, 2009). The mechanisms behind central nervous system sensitization, dysautonomia, and vulvodynia are unclear. The prevalence of these conditions in individuals with HSD/EDS points to the potential of biologic mediators in the nervous system. These mediators may play a role in the development of vulvodynia when an individual undergoes prolonged exposure to a stressor.

### EDS Findings

Our results most closely represent people with hEDS or HSD because of the large percentage of participants with hEDS or HSD (91%). As with vulvodynia the two phenotypes identified may suggest two different clinical presentations of hEDS. The non-musculoskeletal phenotype does not include pain as a defining characteristic even though pain is a diagnostic criterion for hEDS. Rather, the non-musculoskeletal phenotype presents with more non-musculoskeletal symptoms and more systemic conditions. The pain phenotype presents primarily with comorbid conditions associated with joint instability.

Three studies (De Wandele et al., 2013; Petrucci et al., 2024; Schubart et al., 2019) used LCA or cluster analyzes to evaluate comorbid condition patterns in people with EDS but did not include vulvodynia in their analysis. De Wandele et al. (2013) identified three symptom classes; musculoskeletal complaints including pain, non-musculoskeletal complaints, and a co-occurrence of the two clusters. The classes in their analysis were similar to the two overarching phenotypes in this analysis, including the same conditions except for pelvic instability. Petrucci et al. (2024) found three classes, one with the co-occurrence of dysautonomia, gastrointestinal issues, and MCAD, like this analysis. Schubart et al. (2019) reported three classes: high pain, high symptom burden, and mental fatigue. Their phenotypes of high pain and high symptom burden were similar to the two phenotypes in this analysis. De Wanldele et al., 2013; Petrucci et al., 2024; Schubart et al.,2019 and this analysis all identified one class comprised of pain and one that did not include pain or joint related issues, supporting the hypothesis of two presentations of hEDS. Identifying hEDS subtypes typified by non-musculoskeletal conditions stresses the importance of assessing non-musculoskeletal conditions when diagnosing and managing hEDS; this may help identify hEDS in people with the non-musculoskeletal presentation.

### EDS and Vulvodynia

These LCA results highlight the need to consider multiple causative pathways in investigating vulvodynia. We hypothesize that vulvodynia is affected by a person’s allostatic load or “the cost of chronic exposure to fluctuating or heightened neural or neuroendocrine responses resulting from repeated or chronic environmental challenge (McEwen & Stellar, 1993).” We hypothesize that once an allostatic load threshold, unique to each person and possibly influenced by genetics, is crossed, vulvodynia develops, similar to chronic pain where nociceptive pain may cause central nervous system sensitization (Latremoliere & Woolf, 2009). The proposed allostatic load threshold may be met through the accumulation of different physical (Bornstein et al., 2016; Rodriquez et al., 2019), psychological (Bornstein et al., 2016; Rodriquez et al., 2019; Young & Miller, 2019), and environmental stressors (Beese et al., 2022). If the allostatic load drops below the threshold at which vulvodynia develops, symptoms may lessen or resolve.

Allostatic load can be measured with a risk score determined by biomarkers (Rodriquez et al., 2019) such as blood pressure and cortisol levels. Biomarkers are differently correlated with the risk score meaning some biomarkers provide more information than others (Liu et al., 2021). Our analysis demonstrated how distinct patterns of comorbid conditions (phenotypes) differently affected the likelihood of screening vulvodynia+. We hypothesize that each comorbid condition pattern uniquely contributes differently toward the allostatic load and therefore to vulvodynia screening status as well.

Our model suggests comorbid conditions related to pain contribute more toward screening vulvodynia+ than non-musculoskeletal conditions. The indicators in our two phenotypes are in line with current vulvodynia-associated factors and treatment methods. The non-musculoskeletal phenotype is in line with vulvodynia-associated factor of neuroinflammatory proliferation via mast cell activation. A recent symposium of vulvodynia experts has identified neuroinflammatory proliferation as a suspected pathogenesis for vulvodynia and suggest antihistamines as a treatment method including ketotifen fumarate and luteolin. Ketotifen is used to treat mast cell activation syndrome and luteolin is being investigated for efficacy in treating MCAD (Krapf et al., 2022). Whereas the pain phenotype incorporates pelvic floor dysfunction via pelvic instability and central nervous system sensitization and chronic pain conditions via the chronic pain indicator. Most current treatment methods including pelvic floor physical therapy, SNRIs, SSRIs, antiepileptics, lidocaine, acupuncture, botulinum toxin A, and antispasmodics target the pain phenotype (Schlaeger et al., 2023). However, there are fewer treatments that target conditions in the non-musculoskeletal phenotype. Different emerging phenotypes that may develop along different pathways may contribute to what is seen clinically, a lack of consistently effective treatments for vulvodynia.

The relationship between allostatic load and vulvodynia could explain the increased incidence of vulvodynia among people with EDS. The numerous co-morbid conditions and high symptom burden associated with EDS (Volberding et al., 2022) likely leads to an increased allostatic load (Guidi et al., 2020). While the relationship between EDS and allostatic load has not been investigated, EDS comorbid conditions and characteristics such as chronic fatigue syndrome (Guidi et al., 2020; Sibille et al., 2017), dysautonomia (Goldstein, 2020, 2024), fibromyalgia (Guidi et al., 2020), headaches (Castori et al., 2015), mobility disabilities (Hollar, 2013), lower physical function (Mickle et al., 2023), chronic pain (Olsen et al., 2014), and increased pain (Sibille et al., 2017), are all associated with increased allostatic load (Slade & Sanders, 2012). Many of these conditions are also associated with vulvodynia (Bergeron et al., 2020; Bornstein et al., 2016; Harlow et al., 2009). We hypothesize the conditions that increase the likelihood for vulvodynia in people with EDS also increase the likelihood in people without EDS. The relationship between allostatic load and vulvodynia needs further examination.

## Limitations

Cross-sectional self-report surveys are appropriate for examining new phenomena in a large sample. Limitations of this study include not knowing if vulvodynia developed before or after the comorbid conditions and not being able to confirm participants’ diagnoses. This survey’s comorbid condition checklist was not exhaustive, and participants may have forgotten or failed to list conditions. Additionally, generalizability of study findings is limited to the predominantly white sample. Lack of diversity within EDS research is pervasive with most research conducted in predominately white Northern European countries (Glayzer et al., 2024). We attempted to recruit a more diverse sample via global sampling however our sample was still predominantly white. This limits the generalizability to those with EDS who are not white. The lack of diversity among participants may also be due to the lack of healthcare providers that diagnose in countries that are not predominately white, structural inequalities in the United States that limits access to providers that diagnose EDS, the survey being in English, and the platforms where the survey was advertised (Black et al., 2023; Xu et al., 2024). Furthermore, participants may have not been diagnosed with comorbid conditions specific to EDS if their providers are not familiar with them (Halverson et al., 2021). We are currently replicating this analysis using a dataset with a diverse sample and more complete comorbid condition inventory, guided by the findings of this study, and including participants screening vulvodynia+ without EDS. We will compare the findings of the new study with these findings.

A further limitation was that MCAD was mistakenly listed as “MCAD, medium-acyl-CoA dehydrogenase” instead of “MCAD, mast cell activation disorder” in the checklist. Ehlers–Danlos syndromes and medium-acyl-CoA dehydrogenase are both rare. It is unlikely for participants to have been diagnosed with both medium-acyl-CoA dehydrogenase and EDS or HSD. Therefore, with guidance from EDS experts, we combined medium-acyl-CoA dehydrogenase from the checklist with mast cell activation disorder/syndrome from the write-ins. MCAD is a comorbid condition prevalent in EDS and therefore important to include in the analysis despite this limitation.

Vulvodynia can only be diagnosed with a pelvic exam (Schlaeger et al., 2023). We screened for vulvodynia with four reliable and valid questions (Fig. 2). Since the cause(s) of vulvodynia is unknown, it is diagnosed by ruling out other causes of vulvar pain and dyspareunia. Members of the *Other Pain Conditions* class endorsed indicators used in the clinic to rule out vulvodynia, such as lacerations. While possible for participants to have such conditions and vulvodynia, the presence of these conditions often rules out a vulvodynia diagnosis. The co-occurrence of these conditions in participants who screened vulvodynia + may suggest a weakness in the vulvodynia screening tool. Therefore, the tool should be further evaluated.

## Conclusion

Discussing vulvar pain and dyspareunia can be difficult for patients and providers. Understanding which patients may more likely to develop vulvodynia can ensure they are screened and monitored for the development of vulvodynia symptoms. The non-musculoskeletal phenotype incurs the lowest likelihood of screening positive for vulvodynia, followed by the pain phenotype, with the co-occurrence of both phenotypes incurring significantly greater likelihood. The two comorbid condition phenotypes identified, pain and non-musculoskeletal, may be two different phenotypic presentations of vulvodynia and/or EDS and/or represent different pathways for developing vulvodynia. The presence of these phenotypes is supported by prior EDS research (De Wandele et al., 2013; Petrucci et al., 2024; Schubart et al., 2019). Further longitudinal research is needed for empirical validation of these phenotypes. The presence of multiple phenotypes and/or pathways may explain why there are not consistently effective treatments for vulvodynia. These phenotypes may be used to develop or select targeted therapies based on the presenting phenotype. The hypothesis that an increase in allostatic load is associated with vulvodynia is new and suggests a novel paradigm for understanding and treating vulvodynia. This hypothesis should also be explored in future longitudinal studies.

## Supplementary Material

Supplement Materials

Supplementary material associated with this article can be found, in the online version, at doi:10.1016/j.pmn.2026.03.005.

## Figures and Tables

**Table 1:** Characteristics of the Analyzed Sample ( *n* = 1,061)

Demographics	N	Mean ±SD/Range
Age		
Mean	1,016	37.9 ± 11.3
Range	1,016	18-77
	N	**%**
Gender		
Female	1,015	99.9
Transgender	1	.1
Race		
White	939	92.4
Black of African American	12	1.2
American Indian or Alaskan Native	15	1.5
Asian	11	1.1
Native Hawaiian or Pacific Islander	3	0.3
Other	44	4.3
Ethnicity^a^		
Hispanic or Latino	36	3.6
Not Hispanic or Latino	920	90.8
Unknown or not reported	57	45.6
Disabled	149	14.6

Abbreviation: SD = standard deviations.

a Participants selected all races that they identified with.

**Table 2:** Measurement Invariance Testing

Model	# of Classes	# of Parameters	Loglikelihood	AIC	BIC	aBIC
Invariant	5	49	−5,407.73	10,913.45	11,154.71	10,999.08
Variant	5	93	−5,324.61	10,835.21	11,293.11	10,997.73
Difference *x*^2^	2( *l*_1_ – *l*_2_) = 166.63, *df* = 44, *p* < .001					

Abbreviation: AIC = Akaike Information Criterion; BIC = Bayesian Information Criterion; aBIC = sample-size-adjusted Bayesian Information Criterion; *df* = degrees of freedom.

**Table 3:** Model Fit and Selection Information

# of Classes	# of Parameters	Loglikelihood	AIC	BIC	aBIC
3	29	−5445.97	10949.95	11092.73	11000.63
4	39	−5422.76	10923.52	11115.54	10991.68
5	49	−5407.73	10913.45	11154.71	10999.08

Cells with a colored background have a fit criteria value that suggests the model represented in the row is the best fit for the data.

**Table 4:** Parameter Estimates for the Five-Class Model

		Few Comorbidities	Non- Musculoskeletal EDS Comorbidities	Hypermobile EDS Comorbidities	Other Pain Conditions	Numerous Multifaceted Comorbidities
Class Prevalence		0.185 (n=188)	0.183 (n=185)	0.170 (n=173)	0.265 (n=269)	0.198 (n=201)
Indicator	Proportion in Sample					
Dysautonomia	0.73	0.28	0.93	0.80	0.64	1.00
Neck Related Conditions	0.61	0.27	0.64	0.73	0.52	0.91
Deep Penetration Dyspareunia	0.52	0.08	0.37	0.00	0.99	0.88
Chronic Pain	0.47	0.25	0.00	0.76	0.59	0.76
Pelvic Instability	0.36	0.09	0.23	0.51	0.40	0.52
Gastrointestinal Conditions	0.32	0.09	0.38	0.34	0.24	0.58
Vulvar Skin Conditions	0.28	0.14	0.26	0.19	0.41	0.34
Endometriosis	0.25	0.09	0.16	0.09	0.38	0.46
Mast Cell Activation Syndrome	0.19	0.00	0.29	0.20	0.04	0.47
Phenotype		none	Non- Musculoskeletal	Pain	Pain	Non- Musculoskeletal & Pain
Probability of Screening Vulvodynia Positive in Rank Order		5^a,b,c^	4^a,b^	3^a^	2^a^	1
Vulvodynia Positive		0.39	0.50	0.56	0.64	0.73
Vulvodynia Negative		0.61	0.50	0.44	0.36	0.27

Cells with a colored background are endorsed by at least 10% more than the overall sample proportion.

Endometriosis occurred at a large enough frequency to warrant it being its own indicator. Fibroids was included in deep dyspareunia and also occurred at a high frequency but was not included as its own indicator because endometriosis is a pain condition whereas fibroids are generally not associated with pain. Some fibroids can be painful when hit during penetration which is why they were included in the deep dyspareunia category. Pelvic masses like uterine fibroids, ovarian cysts, generally are not associated with dyspareunia and therefore not significant factors in our analysis.

Endometriosis can also cause other types of pain and chronic inflammatory response and occurred at a high frequency.

This is a study that was funded by the NIH and therefore the official designations of race and ethnicity were used.

That is a question of further investigation on its own.

a Significantly different *Numerous Multifaceted Comorbidities p* < .05.

b Significantly different *Other Pain Conditions p* < .05.

c Significantly different *Hypermobility EDS Comorbidities p* < .05.

## Data Availability

The data that support the findings of this study are openly available in Mendeley Data at https://data.mendeley.com/datasets/bx7wph7584/1. The analytic code is available by request.

## Key Practice Points

Two comorbid condition phenotypes were identified in participants with Ehlers–Danlos syndromes, (1) pain conditions and (2) non-musculoskeletal conditions. These phenotypes may increase the likelihood of developing vulvodynia. We hypothesize developing vulvodynia is linked to a person’s allostatic load comprised of stressor such as emotional, physical, environmental, and comorbid conditions.

## References

[R1] Agarwal SK, Chapron C, Giudice LC, Laufer MR, Leyland N, Missmer SA, Singh SS, & Taylor HS (2019). Clinical diagnosis of endometriosis: A call to action. American Journal of Obstetrics and Gynecology, 220(4) 354.e351–354.e312. 10.1016/j.ajog.2018.12.039. 30625295

[R2] Arthur K, Caldwell K, Forehand S, & Davis K (2016). Pain control methods in use and perceived effectiveness by patients with Ehlers–Danlos syndrome: A descriptive study. Disability and Rehabilitation, 38(11), 1063–1074. 10.3109/09638288.2015.1092175. 26497567

[R3] Ashtari S, & Taylor AD (2022). The internet knows more than my physician: qualitative interview study of people with rare diseases and how they use online support groups. Journal of Medical Internet Research, 24(8), Article e39172. 10.2196/39172. 36006679 PMC9459833

[R4] Beese S, Postma J, & Graves JM (2022). Allostatic load measurement: A systematic review of reviews, database inventory, and considerations for neighborhood research. International Journal of Environmental Research and Public Health, 19(24). 10.3390/ijerph192417006. PMC9779615 36554888

[R5] Bennett SE, Walsh N, Moss T, & Palmer S (2021). Understanding the psychosocial impact of joint hypermobility syndrome and Ehlers–Danlos syndrome hypermobility type: A qualitative interview study. Disability and Rehabilitation, 43 (6), 795–804. 10.1080/09638288.2019.1641848. 31318301

[R6] Bergeron S, Reed BD, Wesselmann U, & Bohm-Starke N (2020). Vulvodynia. Nature Reviews Disease Primer, 6(1), 1–21. 10.1038/s41572-020-0164-2. 32355269

[R7] Black WR, Black LL, & Jones JT (2023). Barriers to the diagnosis, care, and management of pediatric patients with Ehlers–Danlos syndrome in the United States: A qualitative analysis. Global Pediatric Health, 10, Article 2333794X231212081. 10.1177/2333794X231212081 PMC10666714 38024462

[R8] Blackburn PR, Xu Z, Tumelty KE, Zhao RW, Monis WJ, Harris KG, Gass JM, Cousin MA, Boczek NJ, & Mitkov MV (2018). Bi-allelic alterations in AEBP1 lead to defective collagen assembly and connective tissue structure resulting in a variant of Ehlers–Danlos syndrome. American Journal of Medical Genetics Part A, 102(4), 696–705. 10.1016/j.ajhg.2018.02.018. PMC5985336 29606302

[R9] Bornstein J, Goldstein AT, Stockdale CK, Bergeron S, Pukall C, Zolnoun D, & Coady D International Society for the Study of Vulvovaginal Disease. (2016). 2015 ISSVD, ISSWSH and IPPS consensus terminology and classification of persistent vulvar pain and vulvodynia. Obstetrics and Gynecology, 127(4), 745–751. 10.1097/AOG.0000000000001359. 27008217

[R10] Bornstein J, Palzur E, Swash M, & Petros P (2022). Vulvodynia: A neuroinflammatory pain syndrome originating in pelvic visceral nerve plexuses due to mechanical factors. Archives of Gynecology and Obstetrics, 306(5), 1411–1415. 35147761 10.1007/s00404-022-06424-4 PMC9519726

[R11] Brock I, Prendergast W, & Maitland A (2021). Mast cell activation disease and immunoglobulin deficiency in patients with hypermobile Ehlers–Danlos syndrome/hypermobility spectrum disorder. American Journal of Medical Genetics Part C: Seminars in Medical Genetics, 187(4), 473–481. 10.1002/ajmg.c.31940. 34747107

[R12] Castori M. (2016). Pain in Ehlers–Danlos syndromes: manifestations, therapeutic strategies and future perspectives. Expert Opinion on Orphan Drugs, 4(11), 1145–1158. 10.1080/21678707.2016.1238302.

[R13] Castori M, Morlino S, Ghibellini G, Celletti C, Camerota F, & Grammatico P (2015). Connective tissue, Ehlers–Danlos syndrome (s), and head and cervical pain. American Journal of Medical Genetics Part C: Seminars in Medical Genetics, 169 (1), 84–96. 10.1002/ajmg.c.31426. 25655119

[R14] Chan C, Krahe A, Lee YT, & Nicholson LL (2019). Prevalence and frequency of self-perceived systemic features in people with joint hypermobility syndrome/Ehlers–Danlos syndrome hypermobility type. Clinical Rheumatology, 38 , 503–511. 10.1007/s10067-018-4296-7. 30232714

[R15] Collins L, & Lanza S (2010). Latent class and latent transition analysis: With applications in the social, behavioral, and health sciences. New Jersey: Wiley & Sons. 10.1002/9780470567333.

[R16] Cox KJ, & Neville CE (2012). Assessment and management options for women with vulvodynia. Journal of Midwifery and Women’s Health, 57(3), 231–240. 10.1111/j.1542-2011.2012.00162.x. 22594863

[R17] De Wandele I, Rombaut L, Malfait F, De Backer T, De Paepe A, & Calders P (2013). Clinical heterogeneity in patients with the hypermobility type of Ehlers–Danlos syndrome. Research in Developmental Disabilities, 34(3), 873–881. 10.1016/j.ridd.2012.11.018. 23291504

[R18] Doherty TA, & White AA (2018). Postural orthostatic tachycardia syndrome and the potential role of mast cell activation. Autonomic Neuroscience, 215, 83–88. 10.1016/j.autneu.2018.05.001. 30033040

[R19] Giesecke J, Reed BD, Haefner HK, Giesecke T, Clauw DJ, & Gracely RH (2004). Quantitative sensory testing in vulvodynia patients and increased peripheral pressure pain sensitivity. Obstetrics and Gynecology, 104(1), 126–133. 10.1097/01.AOG.0000129238.49397.4e. 15229011

[R20] Glayzer JE, Bray BC, Kobak WH, Steffen AD, & Schlaeger JM (2024). Lack of diversity in research on females with Ehlers–Danlos syndromes: recruitment protocol for a quantitative online survey. Journal of Medical Internet Research: Research Protocols, 13 (1), Article e53646. 10.2196/53646. PMC11099804 38696252

[R21] Glayzer JE, McFarlin BL, Castori M, Suarez ML, Meinel MC, Kobak WH, Steffen AD, & Schlaeger JM (2021). High rate of dyspareunia and probable vulvodynia in Ehlers–Danlos syndromes and hypermobility spectrum disorders: an online survey. American Journal of Medical Genetics Part C: Seminars in Medical Genetics, 187(4), 599–608. 10.1002/ajmg.c.31939. 34747110 PMC8665058

[R22] Goldstein AT, Pukall CF, Brown C, Bergeron S, Stein A, & Kellogg-Spadt S (2016). Vulvodynia: assessment and treatment. Journal of Sexual Medicine, 13(4), 572–590. 10.1016/j.jsxm.2016.01.020. 27045258

[R23] Goldstein DS (2020). The extended autonomic system, dyshomeostasis, and Covid-19. Clinical Autonomic Research, 30(4), 299–315. 10.1007/s10286-020-00714-0. 32700055 PMC7374073

[R24] Goldstein DS (2024). Post-covid dysautonomias: what we know and (mainly) what we don’t know. Nature Reviews: Neurology, 20(2), 99–113. 10.1038/s41582-023-00917-9. 38212633

[R25] Guidi J, Lucente M, Sonino N, & Fava GA (2020). Allostatic load and its impact on health: A systematic review. Psychotherapy and Psychosomatics, 90(1), 11–27. 10.1159/000510696. 32799204

[R26] Haefner HK, Collins ME, Davis GD, Edwards L, Foster DC, Hartmann ED, Kaufman RH, Lynch PJ, Margesson LJ, Moyal-Barracco M, Piper CK, Reed BD, Stewart EG, & Wilkinson EJ (2005). The vulvodynia guideline. Journal of Lower Genital Tract Disease, 9 (1), 40–51. https://www.ncbi.nlm.nih.gov/pubmed/15870521. 15870521 10.1097/00128360-200501000-00009

[R27] Halverson CME, Clayton EW, Garcia Sierra A, & Francomano C (2021). Patients with Ehlers–Danlos syndrome on the diagnostic odyssey: Rethinking complexity and difficulty as a hero’s journey. American Journal of Medical Genetics Part C: Seminars in Medical Genetics, 187(4), 416–424. 10.1002/ajmg.c.31935. 34524722

[R28] Harlow BL, Vazquez G, MacLehose RF, Erickson DJ, Oakes JM, & Duval SJ (2009). Self-reported vulvar pain characteristics and their association with clinically confirmed vestibulodynia. Journal of Women’s Health, 18(9), 1333–1340. 10.1089/jwh.2008.1032. PMC2825727 19743906

[R29] Hollar D. (2013). Cross-sectional changes in patterns of allostatic load among persons with varying disabilities, NHANES: 2001–2010. Disability and Health Journal, 6(3), 177–187. 10.1016/j.dhjo.2013.01.009. 23769476

[R30] Hovaguimian A. (2023). Dysautonomia: Diagnosis and management. Neurologic Clinics. 10.1016/j.ncl.2022.08.002. 36400555

[R31] Ji R-R, Nackley A, Huh Y, Terrando N, & Maixner W (2018). Neuroinflammation and central sensitization in chronic and widespread pain. Anesthesiology, 129(2), 343. 10.1097/ALN.0000000000002130. 29462012 PMC6051899

[R32] Krapf JM, Yong PJ, Berke MD, Bohm-Starke N, Bornstein J, Chrysilla E, Dempsey TT, Falsetta ML, Foster D, & Goldstein SW (2022). Executive summary of the vulvodynia therapeutic research summit. Obstetrics and Gynecology, 10, 1097. 10.1097/AOG.0000000000006118 41197126

[R33] Kucharik AH, & Chang C (2020). The relationship between hypermobile Ehlers– Danlos syndrome (hEDS), postural orthostatic tachycardia syndrome (POTS), and mast cell activation syndrome (MCAS). Clinical Reviews in Allergy & Immunology, 58, 273–297. 10.1007/s12016-019-08755-8. 31267471

[R34] Latremoliere A, & Woolf CJ (2009). Central sensitization: A generator of pain hypersensitivity by central neural plasticity. Journal of Pain, 10(9), 895–926. 10.1016/j.jpain.2009.06.012. 19712899 PMC2750819

[R35] Liu SH, Juster RP, Dams-O’Connor K, & Spicer J (2021). Allostatic load scoring using item response theory. Comprehensive Psychoneuroendocrinology, 5, Article 100025. 10.1016/j.cpnec.2020.100025. 35754455 PMC9216382

[R36] Malfait F, Francomano C, Byers P, Belmont J, Berglund B, Black J, Bloom L, Bowen JM, Brady AF, Burrows NP, Castori M, Cohen H, Colombi M, Demirdas S, De Backer J, De Paepe A, Fournel-Gigleux S, Frank M, Ghali N, & …Tinkle B (2017). The 2017 international classification of the Ehlers–Danlos syndromes. American Journal of Medical Genetics Part C: Seminars in Medical Genetics, 175(1), 8–26. 10.1002/ajmg.c.31552. 28306229

[R37] Mathias CJ, Owens A, Iodice V, & Hakim A (2021). Dysautonomia in the Ehlers– Danlos syndromes and hypermobility spectrum disorders—With a focus on the postural tachycardia syndrome. American Journal of Medical Genetics Part C: Seminars in Medical Genetics, 187(4), 510–519. 10.1002/ajmg.c.31951. 34766441

[R38] McEwen BS, & Stellar E (1993). Stress and the individual: Mechanisms leading to disease. Archives of Internal Medicine, 153(18), 2093–2101. 10.1001/archinte.1993.00410180039004. 8379800

[R39] Mickle AM, Tanner JJ, Olowofela B, Wu S, Garvan C, Lai S, Addison A, Przkora R, Edberg JC, & Staud R (2023). Elucidating individual differences in chronic pain and whole person health with allostatic load biomarkers. Brain, Behavior, and Immunity, 33, Article 100682. 10.1016/j.bbih.2023.100682. PMC10493889 37701788

[R40] Morin M, Binik YM, Bourbonnais D, Khalife S, Ouellet S, & Bergeron S (2017). Heightened pelvic floor muscle tone and altered contractility in women with provoked vestibulodynia. Journal of Sexual Medicine, 14(4), 592–600. 10.1016/j.jsxm.2017.02.012. 28364981

[R41] Nguyen RH, Veasley C, & Smolenski D (2013). Latent class analysis of comorbidity patterns among women with generalized and localized vulvodynia: preliminary findings. Journal of Pain Research, 6, 303. 10.2147/JPR.S42940. 23637555 PMC3636807

[R42] Olsen RB, Bruehl S, Nielsen CS, Rosseland LA, Eggen AE, & Stubhaug A (2014). Chronic pain and cardiovascular stress responses in a general population: the tromsø study. Journal of Behavioral Medicine, 37(6), 1193–1201. 10.1007/s10865-014-9568-3. 24793322

[R43] Orhurhu VJ, Roberts JS, Ly N, & Cohen SP (2023, September). 4). Ketamine in acute and chronic pain management. In StatPearls. StatPeals Publishing. Retrieved January 28, 2025 https://europepmc.org/article/NBK/nbk539824. 30969646

[R44] Ossipov MH, Dussor GO, & Porreca F (2010). Central modulation of pain. Journal of Clinical Investigation, 120(11), 3779–3787. 10.1172/JCI43766. 21041960 PMC2964993

[R45] Petrucci T, Barclay SJ, Gensemer C, Morningstar J, Daylor V, Byerly K, Bistran E, Griggs M, Elliot JM, & Kelechi T (2024). Phenotypic clusters and multimorbidity in hypermobile Ehlers–Danlos syndrome. Mayo Clinic Proceedings: Innovations, Quality & Outcomes, 8(3), 253–262. 10.1016/j.mayocpiqo.2024.04.001. PMC11109295 38779137

[R46] Reed BD, Haefner HK, Harlow SD, Gorenflo DW, & Sen A (2006). Reliability and validity of self-reported symptoms for predicting vulvodynia. Obstetrics and Gynecology, 108(4), 906–913. 10.1097/01.AOG.0000237102.70485.5d. 17012453

[R47] Reed BD, Harlow SD, Sen A, Edwards RM, Chen D, & Haefner HK (2012). Relationship between vulvodynia and chronic comorbid pain conditions. Obstetrics and Gynecology, 120(1), 145. 10.1097/AOG.0b013e31825957cf. 22914403 PMC3566870

[R48] Rodriquez EJ, Kim EN, Sumner AE, Nápoles AM, & Pérez-Stable EJ (2019). Allostatic load: importance, markers, and score determination in minority and disparity populations. Journal of Urban Health, 96(Suppl 1), 3–11. 10.1007/s11524-019-00345-5. 30671711 PMC6430278

[R49] Rombaut L, Malfait F, De Paepe A, Rimbaut S, Verbruggen G, De Wandele I, & Calders P (2011). Impairment and impact of pain in female patients with Ehlers–Danlos syndrome: A comparative study with fibromyalgia and rheumatoid arthritis. Arthritis and Rheumatism, 63(7), 1979–1987. 10.1002/art.30337. 21391202

[R50] Rombaut L, Scheper M, De Wandele I, De Vries J, Meeus M, Malfait F, Engelbert R, & Calders P (2015). Chronic pain in patients with the hypermobility type of Ehlers–Danlos syndrome: Evidence for generalized hyperalgesia. Clinical Rheumatology, 34(6), 1121–1129. 10.1007/s10067-014-2499-0. 24487572

[R51] Schlaeger JM, Glayzer JE, Villegas-Downs M, Li H, Glayzer EJ, He Y, Takayama M, Yajima H, Takakura N, & Kobak WH (2023). Evaluation and treatment of vulvodynia: State of the science. Journal of Midwifery and Women’s Health, 68(1), 9–34. 10.1111/jmwh.13456. PMC10107324 36533637

[R52] Schubart JR, Schaefer E, Hakim AJ, Francomano CA, & Bascom R (2019). Use of cluster analysis to delineate symptom profiles in an Ehlers–Danlos syndrome patient population. Journal of Pain and Symptom Management, 58(3), 427–436. 10.1016/j.jpainsymman.2019.05.013. 31153935 PMC6708773

[R53] Si M, Chen J, Zhang X, Zhu L, & Jiang Y (2024). Pain and daily interference among reproductive-age women with myofascial pelvic pain: serial mediation roles of kinesiophobia, self-efficacy and pain catastrophizing. PloS One, 19 (5), Article e0301095. 10.1371/journal.pone.0301095. 38739604 PMC11090321

[R54] Sibille KT, McBeth J, Smith D, & Wilkie R (2017). Allostatic load and pain severity in older adults: results from the English longitudinal study of ageing. Experimental Gerontology, 88, 51–58. 10.1016/j.exger.2016.12.013. 27988258 PMC5326483

[R55] Slade GD, & Sanders AE (2012). Role of allostatic load in sociodemographic patterns of pain prevalence in the US population. Journal of Pain, 13(7), 666–675. 10.1016/j.jpain.2012.04.003. 22677453 PMC3652569

[R56] Theoharides TC, Twahir A, & Kempuraj D (2024). Mast cells in the autonomic nervous system and potential role in disorders with dysautonomia and neuroinflammation. Annals of Allergy, Asthma & Immunology, 132(4), 440–454. 10.1016/j.anai.2023.10.032. 37951572

[R57] Tinkle BT, & Levy HP (2019). Symptomatic joint hypermobility: The hypermobile type of Ehlers–Danlos syndrome and the hypermobility spectrum disorders. Medical Clinics of North America, 103(6), 1021–1033. 10.1016/j.mcna.2019.08.002. 31582002

[R58] Tinkle B, Castori M, Berglund B, Cohen H, Grahame R, Kazkaz H, & Levy H (2017). Hypermobile Ehlers–Danlos syndrome (A.K.A . Ehlers–Danlos syndrome type III and Ehlers–Danlos syndrome hypermobility type): Clinical description and natural history. American Journal of Medical Genetics Part C: Seminars in Medical Genetics, 175(1), 48–69. 10.1002/ajmg.c.31538. 28145611

[R59] Torres-Cueco R, & Nohales-Alfonso F (2021). Vulvodynia—It is time to accept a new understanding from a neurobiological perspective. International Journal of Environmental Research and Public Health, 18(12), 6639. 34205495 10.3390/ijerph18126639 PMC8296499

[R60] Voermans NC, & Knoop H (2011). Both pain and fatigue are important possible determinants of disability in patients with the Ehlers–Danlos syndrome hypermobility type. Disability and Rehabilitation, 33(8), 706–707. 10.3109/09638288.2010.531373. 21077749

[R61] Voermans NC, Knoop H, Bleijenberg G, & van Engelen BG (2010). Pain in Ehlers–Danlos syndrome is common, severe, and associated with functional impairment. Journal of Pain and Symptom Management, 40(3), 370–378. 10.1016/j.jpainsymman.2009.12.026. 20579833

[R62] Volberding P, Spicer CM, Cartaxo T, & Wedge R (2022). Selected heritable disorders of connective tissue and disability. Washington, DC, USA: National Academies Press. 10.17226/26431. 36223440

[R63] Wesselmann U, Bonham A, & Foster D (2014). Vulvodynia: Current state of the biological science. Pain, 155(9), 1696–1701. 24858303 10.1016/j.pain.2014.05.010 PMC5568852

[R64] Williams HR, Wood G, Hakim AJ, Birchall M, & Hirani SP (2023). Selfreported throat symptoms in Ehlers–Danlos syndromes and hypermobility spectrum disorders: A cross-sectional survey study. Laryngoscope Investigative Otolaryngology, 8(5), 1259–1264. 10.1002/lio2.1120. 37899864 PMC10601551

[R65] Woolf CJ (2011). Central sensitization: implications for the diagnosis and treatment of pain. Pain, 152(3), S2–S15. 10.1016/j.pain.2010.09.030. 20961685 PMC3268359

[R66] Wu W, & Ho V (2024). An overview of Ehlers–Danlos syndrome and the link between postural orthostatic tachycardia syndrome and gastrointestinal symptoms with a focus on gastroparesis. Frontiers in Neurology, 15, Article 1379646. 10.3389/fneur.2024.1379646. 39268060 PMC11390471

[R67] Xu K, Li G, Wu Z, Zhang TJ, & Wu N Chinese Multi-Disciplinary Working Group on the Ehlers–Danlos Syndromes. (2024). Diagnosis and treatment of the Ehlers–Danlos syndromes in china: Synopsis of the first guidelines. Orphanet Journal of Rare Diseases, 19 (1), 194. 38741208 10.1186/s13023-024-03121-0 PMC11092078

[R68] Young AL, & Miller AD (2019). This girl is on fire” sensemaking in an online health community for vulvodynia. In Proceedings of the 2019 CHI Conference on Human Factors in Computing Systems (pp. 1–13). Glasgow, Scotland, UK.

